# Association of cardiovascular health with morbidity and mortality among U.S. adults with osteoarthritis: a population-based study

**DOI:** 10.1186/s12889-025-22530-9

**Published:** 2025-04-30

**Authors:** Xuan Zhang, Haoxian Tang, Jingtao Huang, Hanyuan Lin, Qinglong Yang, Nan Luo, Jian Weng, Hui Zeng, Fei Yu

**Affiliations:** 1 Department of Bone & Joint Surgery, Peking University Shenzhen Hospital, No. 1120 Lianhua Road, Shenzhen, 518036 Guangdong China; 2 National & Local Joint Engineering Research Center of Orthopaedic Biomaterials, Shenzhen, Guangdong China; 3 Shenzhen Key Laboratory of Orthopaedic Diseases and Biomaterials Research, Shenzhen, Guangdong China; 4 Shantou University Medical College, Shantou, Guangdong China; 5 Department of Cardiology, The First Affiliated Hospital of Shantou University Medical College, Shantou, Guangdong China; 6 Department of Orthopedic Trauma, Shenzhen Second People’s Hospital, the First Affiliated Hospital of Shenzhen University, Shenzhen, Guangdong China; 7 Department of Spine Surgery, Shenzhen Second People’s Hospital, the First Affiliated Hospital of Shenzhen University, Shenzhen, Guangdong 518035 China

**Keywords:** Cardiovascular health, Life’s essential 8, Osteoarthritis, Mortality, NHANES

## Abstract

**Background:**

Osteoarthritis (OA) is recognized as the most common joint disease with serious public health implications. Cardiovascular health (CVH) is also an issue that is frequently emphasized in public health and has an impact on a variety of diseases and mortality rates. This study aims to investigate the association of CVH with the morbidity of OA. And explore the association of CVH with both all-cause and cardiovascular disease (CVD) mortality among US adults with OA.

**Methods:**

This study utilized data from the National Health and Nutrition Examination Survey 2005–2018, which included 21,289 adults aged ≥ 20, representing 137,912,968 Americans. CVH was assessed by Life’s Essential 8 (LE8) includes 4 behavior and 4 factor metrics. Total LE8 scores were calculated from the unweighted average on a 0–100 scale and were categorized as high (80–100), moderate (50–79), and low (0–49) CVH. Multivariable logistic regression explored the association of OA with CVH. Cox proportional hazards regression examined LE8 associations with mortality.

**Results:**

Adjusting for confounding variables, per 10 points LE8 increase, the OR was 0.82 in association with OA, while OA morbidity were decreased by 30% (OR 0.70, 95%CI 0.57, 0.87) and 54% (OR 0.46, 95%CI 0.36, 0.60) in moderate and high CVH compared to low CVH. During a median follow-up of 7.58 years in OA participants, per 10 points LE8 increase were decreased 23% mortality of all-cause (HR 0.77, 95%CI 0.70, 0.85) and 29% mortality of CVD (HR 0.71, 95% 0.60, 0.84). Moderate and high CVH demonstrated a decreased mortality of both all-cause and CVD compared with low CVH.

**Conclusions:**

Higher CVH is associated with a lower morbidity of OA and lower mortality in OA participants. Our results suggest that adherence to CVH could reduce the morbidity of OA and improve survival outcomes for those affected.

**Supplementary Information:**

The online version contains supplementary material available at 10.1186/s12889-025-22530-9.

## Introduction

Osteoarthritis (OA), acknowledged as the foremost prevalent joint disorder, is characterized by degenerative changes in articular cartilage and secondary periarticular osteophytes [1]. It impacted approximately 520 million individuals worldwide in 2019, marking a substantial 113% increase from the figures recorded in 1990 [2]. The advancement of OA leads to pain and limitations in daily activities, detrimentally impacting patients'quality of life and serving as a significant contributor to disability [3]. The absence of effective medications to arrest the progression of OA underscores the reliance on pain relief and lifestyle interventions as primary treatments, and the substantial prevalence of OA and its consequential adverse outcomes contribute significantly to the medical economic burden [4, 5].

Risk factors for OA encompass older age, obesity, genetics, joint injuries, and poor lifestyle. The prevalent strategy in OA prevention involves mitigating exposure to these risk factors, with particular emphasis on modifiable elements such as diet, physical activity (PA), and sedentary behavior [6]. Studies have substantiated significant associations between OA and various comorbidities, placing particular emphasis on cardiovascular diseases (CVD) [7, 8]. Furthermore, the mortality rate in OA varies based on specific comorbidities [9–11]. There are shared risk factors between CVD and OA (i.e., sedentary behavior, PA, obesity). These may therefore offer a mechanism of co-morbidity between CVD and OA [12, 13]. Consequently, we propose and aim to investigate the potential role that maintaining cardiovascular health (CVH) may play in the occurrence of OA and the well-being of OA patients.

The Life’s Essential 8 (LE8), proposed by the American Heart Association (AHA), serves as an updated method for evaluating CVH. This scoring system encompasses health behaviors (diet, PA, nicotine exposure, sleep health) and health factors (body mass index [BMI], blood lipids, blood glucose, blood pressure [BP]) [14, 15]. LE8 exhibits sensitivity to both inter-individual variations and temporal changes at both individual and population, provides a comprehensive and sensitive assessment of CVH.

Previous studies have demonstrated a dearth of investigations into the correlation between CVH and OA, despite some elucidation of certain LE8 components (e.g., diet, smoking, and sleep) in relation to OA [16–18]. Additionally, systematic exploration of the correlation between CVH and OA, as well as the association of CVH with all-cause and CVD mortality in individuals with OA, remains relatively underexplored. Given that LE8 is a novel and important measure of CVH, investigating its relationship with OA and mortality in OA patients is therefore essential.

Consequently, the purpose of this study was to investigate the association between CVH assessed by LE8 and the morbidity of OA by analyzing the National Health and Nutrition Examination Survey (NHANES) in a large representative sample data from 2005 to 2018. Additionally, we further investigate the association of LE8 with all-cause and CVD mortality among OA patients in the United States.

## Methods

### Data sources

NHANES constitutes a sequence of surveys conducted by the National Center for Health Statistics (NCHS) to assess the health and nutritional status of a representative segment of the noninstitutionalized U.S. population, utilizing a complex, multistage probability cluster design for data collection and study methodology [19]. Blood samples were collected at the mobile examination center (MEC). All NHANES participants provided written informed consent, and the survey protocol obtained approval from the NCHS Research Ethics Review Board.

### Study design and population

**Fig. 1:**
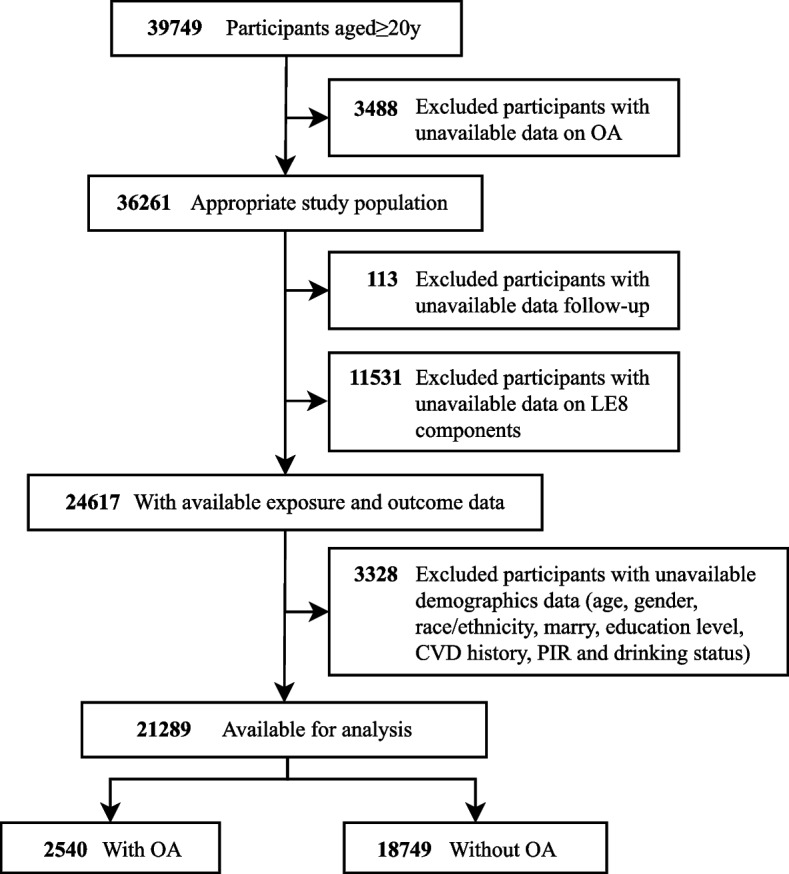
Flowchart of the study population

This study employed 7 consecutive cycles of NHANES data covering the years 2005 to 2018 (2005–2006, 2007–2008, 2009–2010, 2011–2012, 2013–2014, 2015–2016, 2017–2018) and included a total of 39,749 adults aged ≥ 20. Exclusion criteria were applied, leading to the removal of individuals with missing data on OA, follow-up, as well as LE8 components. Additionally, participants with incomplete demographic information, including age, sex, race/ethnicity, marital status, education level, history of CVD, poverty income ratio (PIR), and drinking status, were further excluded. Ultimately, our complete analysis encompassed 21,289 participants (Fig. 1). This study was conducted in accordance with the STROBE guidelines.

### Assessment of life’s essential 8

The CVH scoring algorithm measured by LE8 includes 4 health behaviors metrics (diet, PA, nicotine exposure, and sleep health) and 4 health factors metrics (BMI, blood lipids, blood glucose, and BP). The total score for LE8 is calculated from the unweighted average of 8 CVH metrics and each of them is scored on a scale ranging from 0 to 100 points. Detailed algorithms for calculating the LE8 scores for each metric can be referenced in Supplementary Table 1 and have been previously published, and participants were categorized into high, moderate, and low CVH levels based on LE8 scores of 80 to 100, 50 to 79, and 0 to 49, respectively [14, 15]. The Healthy Eating Index (HEI)− 2015 score was formulated and computed by amalgamating participant dietary intake obtained from two 24-h dietary recalls with food pattern equivalent data from the United States Department of Agriculture (USDA) to assess the dietary metric, and was calculated using the straightforward HEI scoring algorithm method (on a per-person basis) with the assistance of an official SAS code provided by the National Cancer Institute [20, 21]. Frequency and duration of vigorous or moderate-intensity PA in the last 30 days were collected through a self-completed survey. Self-report questionnaires were employed to gather data on nicotine exposure, sleep, history of diabetes mellitus (DM), and the use of medication. Physical examinations were conducted by trained health technicians who measured height, weight, and BP. BMI was determined by dividing the weight in kilograms by the square of the height in meters. Blood lipids, plasma glucose, and hemoglobin A1c (HbA1c) levels were collected and sent to central laboratories for analysis.

### Diagnosis of osteoarthritis

The prevailing case definition commonly employed in epidemiological studies involves self-reported OA diagnosed by doctors, with data regarding the outcome obtained through a questionnaire. Existing research indicates a considerable level of concordance between self-reported and clinically defined OA [22]. Each participant affirmatively responding to the question “Has a doctor or other health professional ever told you that you had arthritis?” underwent a subsequent inquiry: “Which type of arthritis was it?” Those selecting “Osteoarthritis” (2005–2010) or “Osteoarthritis or degenerative arthritis” (2011–2018) were diagnosed with OA.

### Assessment of all-cause and CVD mortality

Mortality information was extracted by the NCHS from death certificate records in the National Death Index [23]. Participants in NHANES 2005–2018 were prospectively followed from the date of enrollment until December 31, 2019 [24]. The International Classification of Diseases, 10 th Revision (ICD- 10) was employed to retrieve and analyze data for both all-cause mortality and CVD mortality, while CVD mortality was specifically defined as heart diseases (ICD- 10 codes, 100–109, 111, 113, 120–151).

### Covariates

Informed by published research and clinical judgment, several variables were deemed potential confounders, encompassing age, sex, race/ethnicity, marry status, education level, CVD history, PIR, and drinking status [15, 25, 26]. Age was categorized into 20–60, and ≥ 60 years. Sex, race/ethnicity were selected from fixed categories of self-reported. Race/ethnicity were classified into Mexican American, Non-Hispanic Black, Non-Hispanic White, Other Hispanic, and Others. Education level was divided into three categories: less than high school, high school or equivalent, and above high school. Self-reported CVD history was recorded if the participant had previously been diagnosed with heart failure, coronary heart disease, angina, heart attack, or stroke. The PIR was categorized into ≤ 1.30, 1.31–3.50, and > 3.50. Self-reported drinking status was categorized as follows: never (consumed < 12 drinks in a lifetime), former (consumed ≥ 12 drinks in one year but did not drink in the last year, or did not drink in the last year but consumed ≥ 12 drinks in a lifetime), mild (female drinking ≤ 1 and male drinking ≤ 2 per day), moderate (female drinking ≤ 2 and male drinking ≤ 3 per day), or heavy (female drinking ≥ 3 and male drinking ≥ 4 per day) [27].

### Statistical analysis

Consistent with NHANES analytic guidelines, we integrated the complex sampling design and the mobile examination center exam sample weights, and the characteristics were delineated concerning CVH levels. Means and standard errors (SE) were used for the presentation of continuous variable data, and information regarding categorical variables was conveyed in terms of numerical counts and percentage frequencies (%). Chi-squared test with Rao & Scott's second-order correction was applied to analyze categorical data, when Wilcoxon rank-sum test for complex survey samples was employed for continuous variables for the evaluation of divergent disparities among various CVH levels. Employing the reverse Kaplan–Meier method, we computed the median follow-up time and its corresponding confidence interval. The calculation extended to determining the number of person-years from the inception of the study to either the event of death or the conclusion of the follow-up period.

CVH score was incorporated into the analysis as both as a continuous variable, with odds ratios (OR) or hazard ratios (HR) computed per 10 points increment, and as a categorical variable categorized into low, moderate, and high CVH levels. We employed multivariable logistic regression models to examine the association between LE8 score and OA. Model 1 was the crude model, did not account for any covariates. Model 2 was adjusted for age, sex, race/ethnicity, marital status, education level, CVD history, PIR, and drinking status. Simultaneously, Cox proportional hazards regression analysis was employed to assess the associations of LE8 with all-cause and CVD mortality within the participants with OA. Survival across distinct CVH groups was estimated using Kaplan–Meier survival curves, and the assessment was carried out through a log-rank test.

Secondary analyses involved conducting stratified analyses based on age, sex, race/ethnicity, marry, education level, CVD history, PIR and drinking status in total participants and those with OA. We employed restricted cubic spline (RCS) analysis with 3 knots to assess potential non-linear associations between LE8 and OA. Additionally, we used chained-equation approach to deal with missing values through multiple imputation with 5 replications.

All analyses were conducted using R, version 4.2.2 (R Project for Statistical Computing) and Free Software Foundation statistics software (version 1.9.2), with the survey package (version 4.1–1). Statistical significance was determined based on 2-sided *p*-values < 0.05.

## Results

### Characteristics of the participants

The characteristics of the study population are summarized in Table 1 by CVH level classification. The study comprised a total of 21,289 participants representing 137,912,968 Americans included for analysis. Among them, 2540 participants reported having OA (12.87%). Over the 17, 892.33 person-years of follow-up (median follow-up, 7.67 years, 95% CI: 7.33, 7.92), there were 436 deaths (mortality per 1,000 person-years, 24.37), including 128 deaths related to CVD (mortality per 1,000 person-years, 7.15). The average LE8 score of the study population was 68.95 (SE, 0.24), with participants respectively categorized into low (*n* = 2548), moderate (*n* = 14,357), and high (*n* = 4384) CVH.

**Table 1:** Characteristics by cardiovascular health level based on Life’s Essential 8 scores of the NHANES 2005–2018 participants

Characteristics	Total	Low CVH	Moderate CVH	High CVH	*P* value
Weighted Population, n[in millions]	137.91	13.16	91.42	33.33	
Age, mean (SE), years	46.90 (0.26)	53.29 (0.42)	48.00 (0.27)	41.38 (0.41)	< 0.001
Age, No (%)					< 0.001
< 60	14688 (75.54)	1409 (63.55)	9622 (73.75)	3657 (85.17)	
≥ 60	6601 (24.46)	1139 (36.45)	4735 (26.25)	727 (14.83)	
Sex, No (%)					< 0.001
Female	10,738 (51.09)	1308 (52.50)	6835 (47.88)	2595 (59.34)	
Male	10,551 (48.91)	1240 (47.50)	7522 (52.12)	1789 (40.66)	
Race/ethnicity, No (%)					< 0.001
Mexican American	3169 (7.65)	339 (7.06)	2244 (8.01)	586 (6.91)	
Non-Hispanic Black	4223 (9.69)	721 (15.39)	2968 (10.35)	534 (5.63)	
Non-Hispanic White	9903 (71.22)	1142 (67.97)	6641 (70.84)	2120 (73.54)	
Other Hispanic	1891 (4.88)	214 (4.52)	1283 (4.87)	394 (5.05)	
Other	2103 (6.57)	132 (5.07)	1221 (5.94)	750 (8.87)	
Marital status, No (%)					< 0.001
Married	11,268 (57.18)	1176 (49.33)	7725 (57.81)	2367 (58.56)	
Never married	3887 (17.59)	352 (13.92)	2362 (15.64)	1173 (24.38)	
Living with partner	1743 (7.96)	204 (9.36)	1204 (8.08)	335 (7.07)	
Other	4391 (17.27)	816 (27.39)	3066 (18.46)	509 (9.99)	
Education level, No (%)					< 0.001
Less than high school	4431 (13.23)	841 (24.33)	3104 (14.12)	486 (6.42)	
High school or equivalent	4808 (22.53)	703 (30.56)	3501 (25.13)	604 (12.23)	
Above high school	12,050 (64.23)	1004 (45.12)	7752 (60.75)	3294 (81.35)	
Drinking status, No (%)					< 0.001
Never	2769 (10.14)	294 (9.07)	1788 (9.67)	687 (11.83)	
Former	3356 (12.86)	679 (24.36)	2341 (13.50)	336 (6.57)	
Mild	7390 (37.70)	700 (29.46)	4894 (36.61)	1796 (43.93)	
Moderate	3439 (18.20)	340 (15.77)	2241 (17.24)	858 (21.78)	
Heavy	4335 (21.11)	535 (21.33)	3093 (22.98)	707 (15.88)	
CVD history, No (%)					< 0.001
No	19,173 (92.18)	1957 (79.25)	12,960 (92.05)	4256 (97.66)	
Yes	2116 (7.82)	591 (20.75)	1397 (7.95)	128 (2.34)	
PIR, mean (SE)	3.12 (0.04)	2.42 (0.06)	3.07 (0.04)	3.51 (0.05)	< 0.001
LE8 scores, mean (SE)					
Total CVH score	68.95 (0.24)	42.29 (0.18)	66.27 (0.12)	86.83 (0.11)	< 0.001
Health behaviors score	67.03 (1.26)	39.23 (1.79)	64.65 (0.93)	84.53 (0.90)	< 0.001
Diet score	39.54 (0.46)	20.10 (0.56)	35.12 (0.45)	59.32 (0.65)	< 0.001
Physical activity score	72.78 (0.50)	28.03 (1.15)	71.58 (0.55)	93.73 (0.37)	< 0.001
Nicotine exposure score	71.85 (0.52)	41.98 (1.15)	68.71 (0.54)	92.27 (0.41)	< 0.001
Sleep health score	83.94 (0.29)	66.82 (0.84)	83.18 (0.29)	92.81 (0.32)	< 0.001
Health factors score	70.87 (0.98)	45.35 (1.57)	67.89 (0.80)	89.12 (0.86)	< 0.001
BMI score	61.17 (0.44)	31.42 (0.79)	56.57 (0.40)	85.56 (0.45)	< 0.001
Blood lipids score	64.63 (0.34)	41.93 (0.82)	61.07 (0.38)	83.38 (0.51)	< 0.001
Blood glucose score	86.93 (0.25)	61.81 (0.79)	86.56 (0.26)	97.84 (0.19)	< 0.001
Blood pressure score	70.73 (0.36)	46.24 (0.70)	67.34 (0.39)	89.69 (0.40)	< 0.001
OA, No (%)					< 0.001
No	18,749 (87.13)	2070 (79.73)	12,582 (86.39)	4097 (92.10)	
Yes	2540 (12.87)	478 (20.27)	1775 (13.61)	287 (7.90)	

All means and SEs for continuous variables and percentages for categorical variables were weighted. LE8 scores range from 0 to 100 and were categorized into low CVH (0–49), moderate CVH (50–79), and high CVH (80–100)

*Abbreviations*: *BMI* Body mass index, *CVD* Cardiovascular Disease, *CVH* Cardiovascular Health, *LE8* Life’s Essential 8, *NHANES* National Health and Nutrition Examination Survey, *OA* Osteoarthritis, *PIR* Poverty Income Ratio, *SE* Standard Error

### Association between the LE8 and OA

**Fig. 2:**
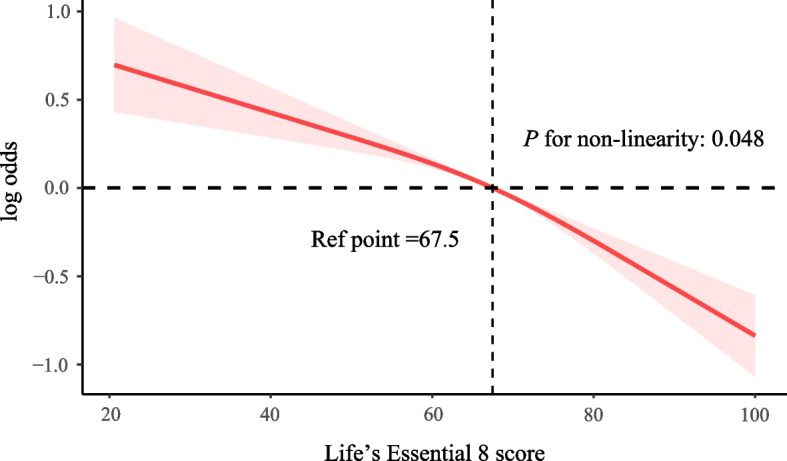
Restrictive cubic spline regression on the association of life’s essential 8 with osteoarthritis of the NHANES 2005–2018 participants. Abbreviations: CVD, cardiovascular disease; PIR, poverty income ratio. Adjusted for age, gender, race/ethnicity, marital status, education level, drinking status, CVD history, PIR

As shown in Table 2, per 10 points increase in the LE8 score was associated with an OR of 0.77 (95% CI = 0.74, 0.81) in association to OA. After adjusting for confounding variables, this association remained and with an OR of 0.82 (95% CI = 0.78, 0.87). In comparison to participants with low CVH, the morbidity of OA was decreased in those with moderate CVH (OR = 0.70, 95% CI = 0.57, 0.87) and high CVH (OR = 0.46, 95% CI = 0.36, 0.60) after adjusting for confounding variables, with *P* for trend < 0.001. Additionally, the analysis of the RCS is depicted in Fig. 2, indicating no linear correlation between the LE8 score and OA after adjusting for confounding variables (*P* for non-linearity: 0.048). Moreover, after adjusting for confounding variables, when the LE8 was divided into health behaviors and health factors, the decreased morbidity remained of per 10 points increase in both health behaviors (OR = 0.94, 95% CI = 0.91, 0.98) and health factors (OR = 0.85, 95% CI = 0.82, 0.88) scores with OA. Further analysis of various LE8 components, the nicotine exposure, BMI, blood glucose and BP were associated with a decreased morbidity of OA. Additionally, the associations above remain significant after multiple imputation for missing variables (Supplementary Table 6).

**Table 2:** Association between cardiovascular health and osteoarthritis of the NHANES 2005–2018 participants

	**Model 1**^**a**^		**Model 2**^**b**^	
	**OR (95%CI)**	***P* value**	**OR (95%CI)**	***P* value**
Total CVH score^c^	0.77 (0.74,0.81)	< 0.001	0.82 (0.78,0.87)	< 0.001
Subgroups^d^				
Low CVH	1 [Reference]		1 [Reference]	
Moderate CVH	0.62 (0.51,0.75)	< 0.001	0.70 (0.57,0.87)	0.002
High CVH	0.34 (0.27,0.42)	< 0.001	0.46 (0.36,0.60)	< 0.001
Trend test		< 0.001		< 0.001
Health behaviors score^e^	0.99 (0.96,1.02)	0.424	0.94 (0.91,0.98)	0.001
Diet score	1.05 (1.03,1.07)	< 0.001	0.98 (0.96,1.00)	0.053
Physical activity score	0.94 (0.93,0.96)	< 0.001	0.99 (0.97,1.00)	0.048
Nicotine exposure score	1.02 (1.00,1.03)	0.013	0.97 (0.96,0.99)	0.003
Sleep health score	1.05 (1.03,1.07)	< 0.001	0.98 (0.96,1.00)	0.053
Health factors score^e^	0.76 (0.73,0.78)	< 0.001	0.85 (0.82,0.88)	< 0.001
BMI score	0.92 (0.90,0.93)	< 0.001	0.90 (0.88,0.91)	< 0.001
Blood lipids score	0.94 (0.92,0.96)	< 0.001	0.98 (0.96,1.00)	0.051
Blood glucose score	0.87 (0.85,0.89)	< 0.001	0.96 (0.94,0.98)	< 0.001
Blood pressure score	0.85 (0.84,0.87)	< 0.001	0.97 (0.94,0.99)	0.010

*Abbreviations*: *BMI* Body mass index, *CVD* Cardiovascular disease, *CVH* Cardiovascular Health, *CI* Confidence interval, *NHANES* National Health and Nutrition Examination Survey, *OR* Odd Ratio, *PIR* Poverty income ratio

^a^Crude model

^b^Adjusted for age, sex, race/ethnicity, marital status, education level, CVD history, PIR, and drinking status

^c^Total CVH score was treated as a continuous variable with per 10 points increase

^d^CVH scores range from 0 to 100 and were categorized into low CVH (0–49), moderate CVH (50–79), and high CVH (80–100)

^e^Health behaviors score (comprising diet score, physical activity score, nicotine exposure score and sleep health score) and health factors score (comprising BMI score, blood lipids score, blood glucose score, and blood pressure score) was treated as a continuous variable with per 10 points increase

### Association of LE8 with all-cause and CVD Mortality in participants with OA

**Fig. 3:**
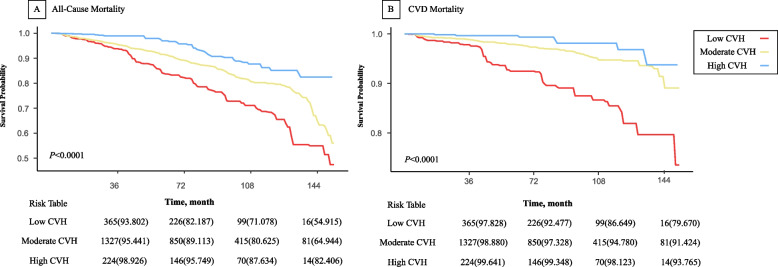
Kaplan–Meier survival curve for All-Cause and CVD mortality of participants with osteoarthritis by life’s essential 8 subgroups

As shown in Table 3, after adjusting for confounding variables, per 10 points increase in LE8 score were decreased 23% of all-cause (HR = 0.77, 95% CI = 0.70, 0.85) and 29% of CVD (HR = 0.71, 95% CI = 0.60, 0.84) mortality. Moreover, compared with participants with low CVH, both all-cause and CVD mortality were decreased in those with moderate (HR = 0.58, 95% CI = 0.43, 0.79, all-cause mortality; HR = 0.34, 95% CI = 0.21, 0.56, CVD mortality) and high (HR = 0.40, 95% CI = 0.25, 0.64, all-cause mortality; HR = 0.27, 95% CI = 0.11, 0.66, CVD mortality) CVH, with *P* for trend < 0.0001. As shown in Supplementary Table 7, the associations above remained significant after multiple imputation for missing variables. Kaplan–Meier survival curve for all-cause and CVD mortality of participants with OA by LE8 subgroups was illustrated in Fig. 3.

**Table 3:** Association of cardiovascular health with all-cause and cardiovascular disease mortality in participants with osteoarthritis of the NHANES 2005–2018 participants

		**Model 1**^**b**^		**Model 2**^**c**^	
	**Events (incidence**^**a**^**)**	**HR (95%CI)**	***P* value**	**HR (95%CI)**	***P* value**
**All-Cause Mortality**					
Total CVH score^d^	436 (24.37)	0.77 (0.70,0.84)	< 0.001	0.77 (0.70,0.85)	< 0.001
Subgroups^e^					
Low CVH	109 (33.95)	1 [Reference]		1 [Reference]	
Moderate CVH	297 (23.52)	0.57 (0.42,0.78)	< 0.001	0.58 (0.43,0.79)	< 0.001
High CVH	30 (14.60)	0.34 (0.21,0.55)	< 0.001	0.40 (0.25,0.64)	< 0.001
Trend test			< 0.001		< 0.001
**CVD Mortality**					
Total CVH score^d^	128 (7.15)	0.69 (0.61,0.79)	< 0.001	0.71 (0.60, 0.84)	< 0.001
Subgroups^e^					
Low CVH	42 (13.08)	1 [Reference]		1 [Reference]	
Moderate CVH	78 (6.18)	0.32 (0.21,0.49)	< 0.001	0.34 (0.21, 0.56)	< 0.001
High CVH	8 (3.89)	0.19 (0.08,0.43)	< 0.001	0.27 (0.11, 0.66)	0.004
Trend test			< 0.001		< 0.001

*Abbreviations*: *CI* Confidence interval, *CVD* Cardiovascular disease, *CVH* Cardiovascular Health, *HR* Hazard Ratios, *NHANES* National Health and Nutrition Examination Survey, *PIR* Poverty income ratio

^a^Number of deaths per 1,000 person-years

^b^Crude model

^c^Adjusted for age, sex, race and ethnicity, marital status, education level, CVD history, PIR, and drinking status

^d^Total CVH score was treated as a continuous variable with per 10 points increase

^e^CVH scores range from 0 to 100 and were categorized into low CVH (0–49), moderate CVH (50–79), and high CVH (80–100)

### Subgroup analyses

Subgroup analysis was performed based on age, sex, race/ethnicity, marital status, education level, CVD history, PIR, and drinking status to evaluated the association of LE8 with OA in total participants (Supplementary Table 3) and the association of LE8 with all-cause (Supplementary Table 4) and CVD (Supplementary Table 5) mortality in participants with OA. In total participants, the interaction was observed between per 10 points increase in the LE8 score and OA in age (*P* for interaction < 0.001) and sex (*P* for interaction = 0.03) subgroups. In participants with OA, in exception of marital status that had an interaction effect in the association between LE8 and all-cause mortality (*P* for interaction = 0.01), no interaction was observed in the remaining subgroups.

## Discussion

In this study, following the adjustment for confounding variables, per 10 points increase in LE8 score was associated with a 18% decreased morbidity of OA, while the morbidity of OA was reduced by 30% and 54% in individuals with moderate and high CVH compared to those with low CVH. In participants with OA, per 10 points increase in LE8 score were decreased 23% mortality of all-cause and 29% mortality of CVD. Compared with participants with low CVH, both all-cause and CVD mortality were decreased in those with moderate and high CVH. The results remain robust after multiple imputation for missing variables.

Specific components of the LE8 have been scrutinized in prior research for their association with OA. Katz et al. noted that individuals with OA exhibited heightened levels of sedentary behavior compared to those without OA, and diminished PA was linked to a 20% increase in age-adjusted mortality rates [28]. Wang et al. revealed that a higher pro-inflammatory potential of the diet was correlated with an elevated risk of OA among older adults in the US [29]. Furthermore, two Mendelian randomization analyses had further disclosed independent harmful causal effects of both smoking and unhealthy sleep on the risk of OA [30, 31].

In addition to the previously discussed LE8 metrics, Kulkarni et al. observed that OA contributed to weight gain [32]. Concurrently, animal experiments suggest that paracrine signaling from adipose tissue may play a pivotal role in the pathophysiology of OA as a mediator of joint degeneration [33]. Furthermore, studies based on the UK Biobank confirm the causal association of circulating lipids with OA [34]. DM has been recognized as an independent risk factor for OA, early alterations in the synovial membrane and subchondral bone in OA, considered a systemic disease, are strongly linked to obesity and metabolic syndrome, with these associations mediated through systemic mechanisms related to DM [35, 36]. A secondary analysis of data from participants in a randomized controlled trial conducted by the Multicenter Study found a correlation between blood pressure and the progression of knee OA [37]. The results of the studies mentioned above are corroborating our findings. More importantly, patients with OA were at increased risk of death from CVD [9], revealing a role for CVH in reducing mortality in the OA population.

The LE8 components, including BP, blood lipids and blood glucose, believed to be linked to CVD and exhibit a strong correlation with both the onset and progression of OA. In previous investigations, the vascular aetiology hypothesis has elucidated the impact of hypertension on OA [38]. Specifically, hypertension disrupts the homeostasis of the osteoarticular environment at both biophysical and biochemical levels, resulting in elevated intraosseous pressure and hypoxia. This, in turn, induces bone remodeling at corresponding sites. Concurrently, the activation of the renin-angiotensin system and the endothelin system locally affects the Wnt-β-catenin protein signaling pathway, contributing to cartilage degradation [38]. Considering that BP is a pivotal factor in CVH assessment, it serves as compelling evidence that CVH risk plays a role in OA pathogenesis. Furthermore, in the context of comprehending the noteworthy positive correlation between 25-hydroxyvitamin D and OA [39], it is intriguing to note that serum 25-hydroxyvitamin D concentrations impact serum high-density lipoprotein (HDL) concentrations [40]. HDL shows a positive correlation with serum 25-hydroxyvitamin D concentrations in the mentioned study. In essence, a high HDL proportion correlates with lower LE8 scores and a less favorable CVH status, which suggests that a heightened prevalence of OA might be linked to lower LE8 scores in this manner [26]. A substantial intake of 25-hydroxyvitamin D has been shown to elevate blood pressure and influence glucose metabolism, primarily through the activation of the renin–angiotensin–aldosterone system and the modulation of systemic inflammatory pathways [41, 42]. Finally, a higher incidence among individuals with a high BMI [39], underscores the onset of OA can affect LE8 scores through BMI changes.

To the best of our knowledge, this study represents the first investigation within a large and representative sample examining the association between CVH assessed by LE8 and the morbidity of OA, and explores the association of LE8 with all-cause and CVD mortality in the OA population. The LE8 provides a comprehensive measurement and sensitive scoring system that and granularly describes CVH, and the rigorous quality control procedures used by NHANES in data collection, as well as its sophisticated sampling design, allowed us to assess the association between LE8 and the risk of OA in a large, representative sample of adults in the United States.

However, the present study has several limitations. Firstly, given its observational nature, establishing definitive cause-and-effect relationships is currently unfeasible. Confounding effects stemming from measurement errors or residuals from unmeasured variables or unknown confounders cannot be entirely discounted. Secondly, the absence of follow-up data in the NHANES dataset poses challenges in evaluating participants'ongoing LE8 status. Incorporating additional follow-up data, enriching the analysis cohort, or employing randomized controlled trial (RCT) study designs could enhance our understanding of LE8 dynamics. Lastly, the diagnosis of OA and certain data elements in LE8, such as PA and sleep, hinge on self-reporting, which is prone to measurement errors and data collection inaccuracies. Employing scientifically robust tools or modalities could enhance the objectivity of the pertinent data and their inherent outcomes.

In conclusion, higher CVH is associated with a lower morbidity of OA and lower mortality in participants with OA. Our results suggest that adherence to CVH could potentially reduce the morbidity of OA and improve survival outcomes for those affected.

## Supplementary Information

Supplementary Material 1

## Data Availability

The data derived from the National Health and Nutrition Examination Survey can be publicly accessed at: https://wwwn.cdc.gov/nchs/nhanes.
