# Supplementary material for: Association of cardiovascular health with morbidity and mortality among U.S. adults with osteoarthritis: a population-based study
Source: BMC Public Health. 2025 Apr 30;25:1587. doi: 10.1186/s12889-025-22530-9 (PMC12042568; doi:10.1186/s12889-025-22530-9)
Supplement: Supplementary file 1 — Supplementary Material 1 [file 12889_2025_22530_MOESM1_ESM.docx]

**Supplementary Materials Files**

To: **Association of Cardiovascular Health with Morbidity and Mortality Among U.S. Adults with Osteoarthritis: A Population-Based Study**

by Xuan Zhang, Haoxian Tang, Jingtao Huang, Hanyuan Lin, Qinglong Yang, Jian Weng, Hui Zeng, Fei Yu

**Supplementary Table 1. Methods for evaluating each individual CVH metric.**

**Supplementary Table 2. Components and scoring criteria of Healthy Eating Index-2015.**

**Supplementary Table 3. Association of Life’s Essential 8 with osteoarthritis of the NHANES 2005-2018 participants in different subgroups.**

**Supplementary Table 4. Association of Life’s Essential 8 with all-cause mortality of the NHANES 2005-2018 participants with osteoarthritis in different subgroups.**

**Supplementary Table 5. Association of Life’s Essential 8 with CVD mortality of the NHANES 2005-2018 participants with osteoarthritis in different subgroups.**

**Supplementary Table 6.** **Association between Life’s Essential 8 and osteoarthritis of the NHANES 2005-2018 participants** **after** **multiple imputation.**

**Supplementary Table 7. Association of Life’s Essential 8 with all-cause and CVD mortality in participants with osteoarthritis of the NHANES 2005-2018 participants after multiple imputation.**

**Supplementary Table 1.** **Methods for evaluating each individual CVH metric**

| **CVH metric** | **Measurement method** | **Scoring of CVH metric (points)** | |
| --- | --- | --- | --- |
|  |  | **Points** | **Status** |
| Diet | Quantiles of the HEI-2015 for the general population | 100 | 95th percentile (ideal diet) |
|  |  | 80 | 75th–94th percentile |
|  |  | 50 | 50th–74th percentile |
|  |  | 25 | 25th–49th percentile |
|  |  | 0 | 1st–24th percentile (least ideal quartile) |
| PA | Self-reported weekly minutes of moderate or vigorous PA | 100 | ≥150 minutes |
|  |  | 90 | 120-149 minutes |
|  |  | 80 | 90-119 minutes |
|  |  | 60 | 60-89 minutes |
|  |  | 40 | 30-59 minutes |
|  |  | 20 | 1-29 minutes |
|  |  | 0 | 0 minutes |
| Nicotine exposure | Self-reported tobacco use (current smoking status and history of smoking) or secondhand smoke exposure (Participants were asked “Does anyone in your household smoke”. Secondhand smoke exposure was defined as if participants’ responses are “yes, one household member smokes” or “Yes, more than one household member smokes”) | 100 | Never smoker, without active indoor smoker |
|  |  | 80 | Never smoker, with active indoor smoker |
|  |  | 75 | Former smoker, quit ≥5 y, without active indoor smoker |
|  |  | 55 | Former smoker, quit ≥5 y, with active indoor smoker |
|  |  | 50 | Former smoker, quit 1–<5 y, without active indoor smoker |
|  |  | 30 | Former smoker, quit 1–<5 y, with active indoor smoker |
|  |  | 25 | Former smoker, quit <1 y, or currently using inhaled NDS, without active indoor smoker |
|  |  | 5 | Former smoker quit <1 y, or currently using inhaled NDS, with active indoor smoker |
|  |  | 0 | Current smoker |
| Sleep health | Self-reported average hours of sleep per night | 100 | 7–<9 hours |
|  |  | 90 | 9-<10 hours |
|  |  | 70 | 6–<7 hours |
|  |  | 40 | 5–<6 or ≥10 hours |
|  |  | 20 | 4–<5 hours |
|  |  | 0 | < 4 hours |
| BMI | Body mass index was calculated as weight (kg) divided by squared height (m2)) | 100 | <25 kg/m2 |
|  |  | 70 | 25.0-29.9 kg/m2 |
|  |  | 30 | 30.0-34.9 kg/m2 |
|  |  | 15 | 35.0-39.9 kg/m2 |
|  |  | 0 | ≥ 40.0 kg/m2 |
| Blood lipid | Measurement of plasma total and HDL cholesterol, including the computation of non-HDL cholesterol | 100 | <130 mg/dL(no medication) |
|  |  | 80 | <130(take medication) |
|  |  | 60 | 130–159(no medication) |
|  |  | 40 | 160–189(no medication) or 130–159(take medication) |
|  |  | 20 | 190–219(no medication) or 160–189 (take medication) |
|  |  | 0 | ≥220 or 190–219 (take medication) |
| Blood Glucose | Casual HbA1c (%) | 100 | No history of diabetes and HbA1c <5.7 % |
|  |  | 60 | No diabetes and HbA1c 5.7–6.4% |
|  |  | 40 | Diabetes with HbA1c <7.0 % |
|  |  | 30 | Diabetes with HbA1c 7.0–7.9 % |
|  |  | 20 | Diabetes with HbA1c 8.0–8.9 % |
|  |  | 10 | Diabetes with HbA1c 9.0–9.9 % |
|  |  | 0 | Diabetes with HbA1c ≥10.0 % |
| Blood pressure | Accurately measured systolic and diastolic BPs | 100 | <120/<80(no medication) |
|  |  | 80 | <120/<80(take medication) |
|  |  | 75 | 120–129/<80 (no medication) |
|  |  | 55 | 120–129/<80 (take medication) |
|  |  | 50 | 130–139 or 80–89(no medication) |
|  |  | 30 | 130–139 or 80–89(take medication) |
|  |  | 25 | 140–159 or 90–99(no medication) |
|  |  | 5 | 140–159 or 90–99(take medication) |
|  |  | 0 | ≥160 or ≥100 |

Abbreviations: BMI, body mass index; BP, blood pressure; CVH, cardiovascular health; HbA1c, hemoglobin A1c; HDL, high-density lipoprotein; HEI, Healthy Eating Index; NDS, nicotine-delivery system; NHANES, National Health and Nutrition Examination Surveys; PA, physical activity.

**Supplementary Table 2. Components and scoring criteria of Healthy Eating Index-2015.**

| Component Characteristics | Component | Maximum Score Criteria | Maximum Score | Minimum Score (zero) Criteria |
| --- | --- | --- | --- | --- |
| *Adequacy^a^* | Total Fruit^c^ | ≥0.8 cup equivalents of per 1,000 kcal | 5 | No fruit |
|  | Whole Fruits^d^ | ≥0.4 cup equivalents of per 1,000 kcal |  | No whole fruit |
|  | Total Vegetables^e^ | ≥1.1 cup equivalents of per 1,000 kcal |  | No vegetables |
|  | Greens and Beans^e^ | ≥0.2 cup equivalents of per 1,000 kcal |  | No dark green vegetables or legumes |
|  | Total Protein Foods^e^ | ≥2.5 oz equivalents of per 1,000 kcal |  | No protein foods |
|  | Seafood and Plant Proteins^e,f^ | ≥0.8 oz equivalents of per 1,000 kcal |  | No seafood or plant proteins |
|  | Whole Grains | ≥1.5 oz equivalents of per 1,000 kcal | 10 | No Whole Grains |
|  | Dairy^g^ | ≥1.3 cup equivalents of per 1,000 kcal |  | No Dairy |
|  | Fatty Acids | (PUFAs + MUFAs)/SFAs ≥2.5 |  | (PUFAs + MUFAs)/SFAs ≤1.2 |
| *Moderation^b^* | Refined Grains | ≤1.8 oz equivalents of per 1,000 kcal |  | ≥4.3 oz equivalents of per 1,000 kcal |
|  | Sodium | ≤1.1 g of per 1,000 kcal |  | ≥2.0 g of per 1,000 kcal |
|  | Added Sugars | ≤6.5% of energy |  | ≥26% of energy |
|  | Saturated Fats | ≤8% of energy |  | ≥16% of energy |

Abbreviations: PUFAs, polyunsaturated fatty acids; MUFAs, monounsaturated fatty acids; SFAs, saturated fatty acids.

^a^. Represent the food groups, subgroups, and dietary elements that are encouraged, higher scores reflect higher intakes.

^b.^ Represent the food groups and dietary elements for which there are recommended limits to consumption, higher scores reflect lower intakes.

^c^. Includes 100% fruit juice.

^d^. Includes all forms except juice.

^e^. Includes legumes (beans and peas).

^f^. Includes seafood, nuts, seeds, soy products (other than beverages), and legumes (beans and peas).

^g^. Includes all milk products, such as fluid milk, yogurt, and cheese, and fortified soy beverages.

**Supplementary Table 3. Association of Life’s Essential 8 with osteoarthritis of the NHANES 2005-2018 participants in different subgroups.**

|  | **OR(95%CI)** | ***P* for interaction** |
| --- | --- | --- |
| Age, No. (%) |  | <0.0001 |
| <60 | 0.72(0.67,0.78) |  |
| ≥60 | 0.91(0.85,0.97) |  |
| Sex, No. (%) |  | 0.03 |
| Female | 0.80(0.76,0.85) |  |
| Male | 0.87(0.80,0.95) |  |
| Race/ethnicity, No. (%) |  | 0.2 |
| Mexican American | 0.86(0.73,1.02) |  |
| Non-Hispanic Black | 0.83(0.75,0.91) |  |
| Non-Hispanic White | 0.83(0.78,0.88) |  |
| Other Hispanic | 0.77(0.65,0.91) |  |
| Other | 0.75(0.60,0.94) |  |
| Marital status, No. (%) |  | 0.57 |
| Married | 0.83(0.77,0.89) |  |
| Never married | 0.87(0.77, 0.99) |  |
| Living with partner | 0.71(0.58,0.88) |  |
| Other | 0.84(0.77,0.92) |  |
| Education level, No. (%) |  | 0.87 |
| Less than high school | 0.80(0.72,0.89) |  |
| High school or equivalent | 0.84(0.75,0.95) |  |
| Above high school | 0.83(0.78,0.88) |  |
| CVD history, No. (%) |  | 0.83 |
| No | 0.83(0.78,0.88) |  |
| Yes | 0.89(0.81,0.98) |  |
| PIR, No. (%) |  | 0.98 |
| ≤1.3 | 0.80(0.74,0.88) |  |
| 1.3~3.5 | 0.83(0.77,0.88) |  |
| >3.5 | 0.84(0.77,0.91) |  |
| Drinking status, No. (%) |  | 0.44 |
| Never | 0.88(0.77,0.99) |  |
| Former | 0.86(0.78,0.96) |  |
| Mild | 0.81(0.76,0.87) |  |
| Moderate | 0.82(0.74,0.92) |  |
| Heavy | 0.81(0.68,0.96) |  |

Abbreviations: CI, Confidence interval; CVD, Cardiovascular disease; NHANES, National Health and Nutrition Examination Survey; OR, Odd Ratio; PIR, Poverty index ratio. Total CVH score was treated as a continuous variable with per 10 points increase. Adjusted for age, sex, race/ethnicity, marital status, education level, CVD history, PIR, and drinking status.

**Supplementary Table 4. Association of Life’s Essential 8 with all-cause mortality of the NHANES 2005-2018 participants with osteoarthritis in different subgroups.**

|  | **HR(95%CI)** | ***P* for interaction** |
| --- | --- | --- |
| Age, No. (%) |  | 0.31 |
| <60 | 0.91(0.63,1.32) |  |
| ≥60 | 0.80(0.72,0.88) |  |
| Sex, No. (%) |  | 0.75 |
| Female | 0.79(0.68,0.92) |  |
| Male | 0.73(0.61,0.87) |  |
| Race/ethnicity, No. (%) |  | 0.1 |
| Mexican American | 0.61(0.22,1.68) |  |
| Non-Hispanic Black | 0.78(0.57,1.07) |  |
| Non-Hispanic White | 0.76(0.66,0.87) |  |
| Other Hispanic | 1.11(0.72,1.71) |  |
| Other | 0.69(0.34,1.40) |  |
| Marital status, No. (%) |  | 0.01 |
| Married | 0.72(0.57,0.92) |  |
| Never married | 2.06(0.98,4.33) |  |
| Living with partner | 1.36(0.48,3.85) |  |
| Other | 0.70(0.59,0.84) |  |
| Education level, No. (%) |  | 0.97 |
| Less than high school | 0.73(0.55,0.98) |  |
| High school or equivalent | 0.75(0.54,1.04) |  |
| Above high school | 0.77(0.66,0.89) |  |
| CVD history, No. (%) |  | 0.25 |
| No | 0.73(0.55,0.98) |  |
| Yes | 0.84(0.68,1.03) |  |
| PIR, No. (%) |  | 0.54 |
| ≤1.3 | 0.83(0.69,0.99) |  |
| 1.3~3.5 | 0.83(0.69,0.99) |  |
| >3.5 | 0.69(0.50,0.95) |  |
| Drinking status, No. (%) |  | 0.47 |
| Never | 0.67(0.49,0.93) |  |
| Former | 0.74(0.58,0.94) |  |
| Mild | 0.84(0.71,1.01) |  |
| Moderate | 0.68(0.43,1.08) |  |
| Heavy | 0.83(0.59,1.18) |  |

Abbreviations: CI, Confidence interval; CVD, Cardiovascular disease; NHANES, National Health and Nutrition Examination Survey; OR, Odd Ratio; PIR, Poverty index ratio. Total CVH score was treated as a continuous variable with per 10 points increase. Adjusted for age, sex, race/ethnicity, marital status, education level, CVD history, PIR, and drinking status.

**Supplementary Table 5. Association of Life’s Essential 8 with CVD mortality of the NHANES 2005-2018 participants with osteoarthritis in different subgroups.**

|  | **HR(95%CI)** | ***P* for interaction** |
| --- | --- | --- |
| Age, No. (%) |  | 0.89 |
| <60 | 0.64(0.39,1.05) |  |
| ≥60 | 0.79(0.65,0.97) |  |
| Sex, No. (%) |  | 0.94 |
| Female | 0.77(0.61,0.98) |  |
| Male | 0.70(0.52,0.94) |  |
| Race/ethnicity, No. (%) |  | 0.87 |
| Mexican American | 0.43(0.19,0.95) |  |
| Non-Hispanic Black | 0.66(0.36,1.20) |  |
| Non-Hispanic White | 0.73(0.60,0.89) |  |
| Other Hispanic | 0.74(0.22,2.51) |  |
| Other | 0.61(0.27,1.41) |  |
| Marital status, No. (%) |  | 0.52 |
| Married | 0.77(0.54,1.09) |  |
| Never married | 1.25(0.79,1.99) |  |
| Living with partner | NA^*^ |  |
| Other | 0.69(0.53,0.91) |  |
| Education level, No. (%) |  | 0.12 |
| Less than high school | 0.67(0.42,1.06) |  |
| High school or equivalent | 1.00(0.68,1.46) |  |
| Above high school | 0.65(0.48,0.88) |  |
| CVD history, No. (%) |  | 0.09 |
| No | 0.60(0.47, 0.78) |  |
| Yes | 0.85(0.66,1.10) |  |
| PIR, No. (%) |  | 0.79 |
| ≤1.3 | 0.72(0.53,0.99) |  |
| 1.3~3.5 | 0.75(0.58,0.96) |  |
| >3.5 | 0.76(0.47,1.21) |  |
| Drinking status, No. (%) |  | 0.83 |
| Never | 0.88(0.60,1.29) |  |
| Former | 0.64(0.43,0.93) |  |
| Mild | 0.74(0.50,1.10) |  |
| Moderate | 0.89(0.53,1.50) |  |
| Heavy | 1.60(0.52,4.86) |  |

^*^. Since the number of participants at this level is too little, OR or its confidence interval cannot be calculated. Abbreviations: CI, Confidence interval; CVD, Cardiovascular disease; NHANES, National Health and Nutrition Examination Survey; OR, Odd Ratio; PIR, Poverty index ratio. Total CVH score was treated as a continuous variable with per 10 points increase. Adjusted for age, sex, race/ethnicity, marital status, education level, CVD history, PIR, and drinking status.

**Supplementary Table 6. Association between Life’s Essential 8 and osteoarthritis of the NHANES 2005-2018 participants after multiple imputation.**

|  | **Model 1^a^** | | **Model 2^b^** | |
| --- | --- | --- | --- | --- |
|  | **OR (95%CI)** | ***P* value** | **OR (95%CI)** | ***P* value** |
| Total CVH score^c^ | 0.79 (0.76,0.82) | <0.001 | 0.84 (0.81,0.88) | <0.001 |
| Subgroups^d^ |  |  |  |  |
| Low CVH | 1[Reference] |  | 1[Reference] |  |
| Moderate CVH | 0.62 (0.54,0.70) | <0.0001 | 0.70 (0.60,0.81) | 0.002 |
| High CVH | 0.36 (0.29,0.43) | <0.0001 | 0.49 (0.39,0.61) | <0.001 |
| Trend test |  | <0.0001 |  | <0.001 |
| Health behaviors score^e^ | 0.98 (0.95,1.01) | 0.118 | 0.94 (0.91,0.97) | <0.001 |
| Diet score | 1.05 (1.04,1.07) | <0.001 | 0.98 (0.97,1.00) | 0.052 |
| Physical activity score | 0.95 (0.94,0.96) | <0.001 | 0.99 (0.98,1.00) | 0.052 |
| Nicotine exposure score | 1.02 (1.00,1.03) | 0.041 | 0.98 (0.96,0.99) | 0.009 |
| Sleep health score | 1.05 (1.03,1.06) | <0.001 | 0.98 (0.96,1.00) | 0.024 |
| Health factors score^e^ | 0.78 (0.76,0.80) | <0.001 | 0.87 (0.85,0.89) | <0.001 |
| BMI score | 0.93 (0.91,0.94) | <0.001 | 0.91 (0.90,0.93) | <0.001 |
| Blood lipids score | 0.95 (0.94,0.97) | <0.001 | 0.98 (0.96,1.00) | 0.047 |
| Blood glucose score | 0.90 (0.89,0.91) | <0.001 | 0.96 (0.95,0.99) | 0.001 |
| Blood pressure score | 0.88 (0.86,0.89) | <0.001 | 0.97 (0.95,0.99) | 0.002 |

Abbreviations: BMI, body mass index; CVD, Cardiovascular disease; CVH, Cardiovascular Health; CI, Confidence interval; NHANES, National Health and Nutrition Examination Survey; OR, Odd Ratio; PIR, poverty income ratio.

^a^. Crude model.

^b^. Adjusted for age, sex, race/ethnicity, marital status, education level, CVD history, PIR, and drinking status.

^c^. Total CVH score was treated as a continuous variable with per 10 points increase.

^d^. CVH scores range from 0 to 100 and were categorized into low CVH (0-49), moderate CVH (50-79), and high CVH (80-100).

^e^. Health behaviors score (comprising diet score, physical activity score, nicotine exposure score and sleep health score) and health factors score (comprising BMI score, blood lipids score, blood glucose score, and blood pressure score) was treated as a continuous variable with per 10 points increase.

**Supplementary Table 7. Association of Life’s Essential 8 with all-cause and CVD mortality in participants with osteoarthritis of the NHANES 2005-2018 participants after** **multiple imputation.**

|  | **Model 1^b^** | | **Model 2^c^** | |
| --- | --- | --- | --- | --- |
|  | **HR (95%CI)** | ***P* value** | **HR (95%CI)** | ***P* value** |
| **All-Cause Mortality** |  |  |  |  |
| Total CVH score^d^ | 0.70 (0.68,0.71) | <0.001 | 0.84 (0.82,0.87) | <0.001 |
| Subgroups^e^ |  |  |  |  |
| Low CVH | 1[Reference] |  | 1[Reference] |  |
| Moderate CVH | 0.47 (0.43,0.51) | <0.001 | 0.71 (0.65,0.77) | <0.001 |
| High CVH | 0.18 (0.15,0.21) | <0.001 | 0.49 (0.42,0.58) | <0.001 |
| Trend test |  | <0.001 |  | <0.001 |
| **CVD Mortality** |  |  |  |  |
| Total CVH score^d^ | 0.67 (0.64,0.71) | <0.001 | 0.82 (0.77, 0.87) | <0.001 |
| Subgroups^e^ |  |  |  |  |
| Low CVH | 1[Reference] |  | 1[Reference] |  |
| Moderate CVH | 0.32 (0.21,0.49) | <0.001 | 0.65 (0.53, 0.78) | <0.001 |
| High CVH | 0.19 (0.08,0.43) | <0.001 | 0.39 (0.27, 0.57) | <0.001 |
| Trend test |  | <0.001 |  | <0.001 |

Abbreviations: CI, Confidence interval; CVD, Cardiovascular disease; CVH, Cardiovascular Health; HR, Hazard Ratios; NHANES, National Health and Nutrition Examination Survey; PIR, poverty income ratio.

^a^. Number of deaths per 1,000 person-years

^b^. Crude model.

^c^. Adjusted for age, sex, race and ethnicity, marital status, education level, CVD history, PIR, and drinking status.

^d^. Total CVH score was treated as a continuous variable with per 10 points increase.

^e^. CVH scores range from 0 to 100 and were categorized into low CVH (0-49), moderate CVH (50-79), and high CVH (80-100).
